# Penalty for Switching Implants? Assessing the Learning Curve With a Collarless, Tapered Wedge Cementless Femoral Component

**DOI:** 10.1016/j.artd.2023.101119

**Published:** 2023-03-06

**Authors:** Brian T. Muffly, Jude C. Kluemper, Cale A. Jacobs, David C. Landy, Stephen T. Duncan

**Affiliations:** Department of Orthopaedic Surgery and Sports Medicine, University of Kentucky, Lexington, KY, USA

**Keywords:** Subsidence, Learning curve, THA, Revision risk, Tapered wedge stem

## Abstract

**Background:**

Surgeon learning curve associated with a tapered wedge femoral implant as measured by early femoral component subsidence and 90-day risk of reoperation was evaluated.

**Methods:**

The first 451 patients undergoing primary, cementless total hip arthroplasty by a single, fellowship-trained arthroplasty surgeon with a tapered wedge stem design were retrospectively reviewed. Early radiographic femoral component subsidence during the first 6 weeks postoperatively and 90-day reoperations was recorded.

**Results:**

When stratified by approach, there was no association between date of surgery and femoral component subsidence in the posterior approach (*P*-value for linear trend over time = 0.44). In the direct anterior approach, there was a significant association between date of surgery and early femoral component subsidence (*P*-value for linear trend over time = 0.01). For both approaches, there was an increase in implanted stem size relative to templated stem size over time (*P* < .01 and *P* = .03, respectively). There was no association between the date of surgery and risk of 90-day reoperation (*P* = .45).

**Conclusions:**

In a single surgeon’s initial use of a tapered cementless wedge stem, early femoral component subsidence was not impacted by the surgeon’s learning curve when the posterior approach was utilized. Although subsidence was associated with date of surgery in the direct anterior cohort, this was not associated with increased risk of 90-day reoperation. Should a surgeon adopt a new tapered-wedge stem, these findings suggest that the stem is forgiving both in relation to subsidence and 90-day reoperation risk when appropriate surgical technique is utilized.

## Introduction

Total hip arthroplasty (THA) is well-known to demonstrate excellent survivorship and improvements in patient function [1]. Implant usage in THA has evolved to coincide with advances in implant design. Among these changes is the increasing trend toward utilization of cementless femoral component fixation in primary THA, reported to be performed in over 95% of THAs as recently as 2021 [2]. The modern tapered wedge cementless femoral component design has allowed for improvements in proximal femoral fit and initial stability to reduce micromotion, promote osseointegration, and reduce subsidence. With increasing use of the direct anterior approach, femoral stems that allow for placement through a more minimally invasive approach may come at the cost of achieving proper initial stability [3,4]. Thus, poor initial stability and lack of osseointegration could potentially impact implant longevity and patient functional outcome [5].

As the demand for primary THA is expected to significantly increase in the coming decades, it has become increasingly important to minimize the potential for subsidence and failure that would contribute to the need for revision surgery and its associated high-cost burden [6,7]. Previous studies have evaluated the role of early postoperative weight-bearing/rehabilitation programs, as well as patient-related factors on femoral component subsidence [[8], [9], [10], [11]]. Little evidence exists regarding the influence of surgeon learning curve on early subsidence of a cementless tapered wedge stem design, including that associated with change in hospital implant vendor and/or surgeon adoption of a new femoral component. Thus, the purpose of the study was to evaluate the learning curve associated with a surgeon’s initial use of a tapered wedge femoral implant as it relates to early femoral component subsidence and 90-day risk of reoperation. The authors hypothesized that the magnitude of femoral component subsidence would decrease with surgeon learning curve, but that 90-day reoperation would be independent of surgeon learning curve.

## Materials and methods

### Study design

A retrospective review of 485 consecutive patients who underwent primary press-fit THA by a single, fellowship-trained arthroplasty surgeon at a single institution was conducted. Institutional review board approval was obtained prior to study initiation. Arthroplasties were performed from December 2015 through January 2021. All THAs were performed with a collarless, cementless, tapered wedge design (POLARSTEM, Smith & Nephew Inc, Memphis, TN). This was the surgeon’s first exposure to and initial use of the implant. At the surgeon’s discretion, either a miniposterior or direct anterior surgical approach was utilized. Patients younger than 18 years of age, those without postoperative follow-up radiographs (at 6 weeks postoperatively), and those patients who were not made weight bearing as tolerated immediately postoperatively were excluded. A total of 34 patients were excluded: 24 with insufficient radiographic follow-up, 8 with altered weight-bearing restrictions in the immediate postoperative period, and 2 patients who were younger than 18 years of age. Each patient was deemed a good surgical candidate for cementless femoral stem fixation at the surgeon’s discretion based on preoperative radiographs demonstrating appropriate bone quality and a proximal femoral anatomy suitable for a tapered wedge stem. Each case was templated preoperatively by the senior author (SD). Intraoperative fluoroscopy was utilized in all cases regardless of approach to evaluate femoral stem size, canal fill, stem position, femoral offset, and acetabular cup positioning.

### Data collection

**Figure 1:**
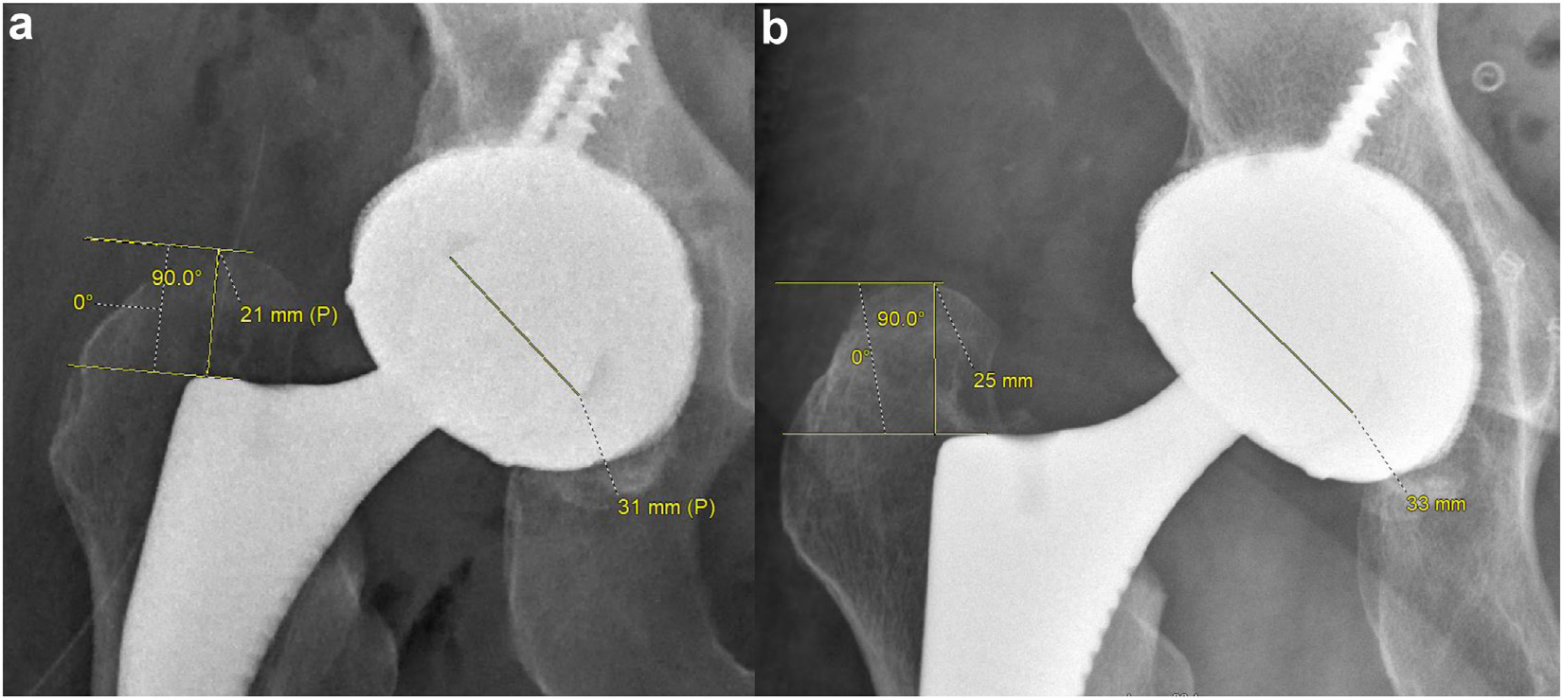
Example of subsidence measurement comparing immediate post-operative radiographs (a) with those taken at 6 weeks postoperatively (b). Known femoral head size was used for magnification correction.

Basic demographic characteristics and surgical data were collected from the electronic medical record. Femoral component subsidence during the first 6 weeks postoperatively and revision procedures within 90 days of the index procedure were recorded. Anteroposterior radiographs were obtained in the recovery room setting and were repeated at either the 2-week or 6-week follow-up visit. Under direction of the senior author (SD) to ensure proper measurement technique, radiographic analysis was performed electronically by a single evaluator (JK) experienced in radiographic measurements of stem subsidence (intraclass correlation coefficient for intraobserver variation >0.95). Using the ruler function of the digital radiographic system (McKesson, San Francisco, CA, USA), all measurements were made as follows: The perpendicular distance between the line tangential to the most prominent aspect of the greater trochanter and the line tangential to the most prominent aspect of superolateral femoral component was calculated [12]. This distance was compared between immediate postoperative radiographs and radiographs obtained at follow-up to determine early femoral component subsidence (Fig. 1). Negative values were set to zero, as the authors believe this finding indicates no movement of the femoral component. The diameter of the femoral head was measured on each film and was used to correct for differences in magnification.

### Statistical analysis

Demographic and clinical characteristics were summarized using descriptive statistics and stratified by surgical approach. Given the clinically significant differences between the characteristics of the patients in different surgical approach groups (Table 1), all further analyses were stratified by surgical approach. This also separates out the potential confounding of approach on other associations.

**Table 1:** Comparison of patient demographic and surgical parameters between surgical approach groups.

Characteristic	Direct anterior	Posterior	*P*
	N = 139	N = 312	
	N (%)	N (%)	
Sex			<.01
Male	89 (64)	138 (44)	
Female	50 (36)	174 (56)	
Age group			.01
<50 years	26 (19)	106 (34)	
50-59 years	53 (38)	104 (33)	
60-69 years	40 (29)	62 (20)	
70-79 years	19 (13)	36 (12)	
80 + years	1 (1)	4 (1)	
BMI category			<.01
<30	88 (63)	132 (42)	
30-34	43 (31)	71 (23)	
35-39	8 (6)	66 (21)	
>40	0 (0)	43 (14)	
CCI group			.14
0-1	78 (56)	206 (66)	
2	39 (28)	59 (19)	
3	20 (15)	41 (13)	
4-6	2 (1)	6 (2)	
Indication			.03
Osteoarthritis	112 (80)	214 (69)	
AVN	19 (14)	54 (17)	
DDH	5 (4)	25 (8)	
Other	3 (2)	19 (6)	

AVN, avascular necrosis; CCI, charlson comorbidity index; DDH, developmental dysplasia of the hip.

The association between date of surgery and subsidence was assessed visually using scatter plots and then empirically assessed using linear regression. For each regression, standardized regression coefficients, Beta, and *P*-values are reported. To assess the association between date of surgery with the difference between the templated and implanted femoral sizes, approach groups were divided into subgroups based upon their date of surgery to create 4 subgroups of similar size for the posterior group and 2 subgroups of similar size for the anterior group. The proportion of cases with various differences between templated and implanted femoral sizes were compared across subgroups using the chi-squared test with *P*-values reported. STATA, version 16 (StataCorp, College Station, TX), was used to perform all statistical analyses.

## Results

**Figure 2:**
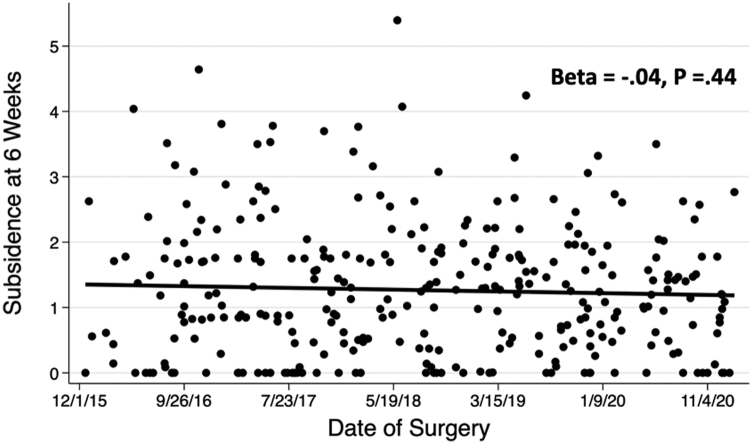
Early femoral component subsidence at 6 weeks postoperatively over time utilizing the posterior approach.

**Figure 3:**
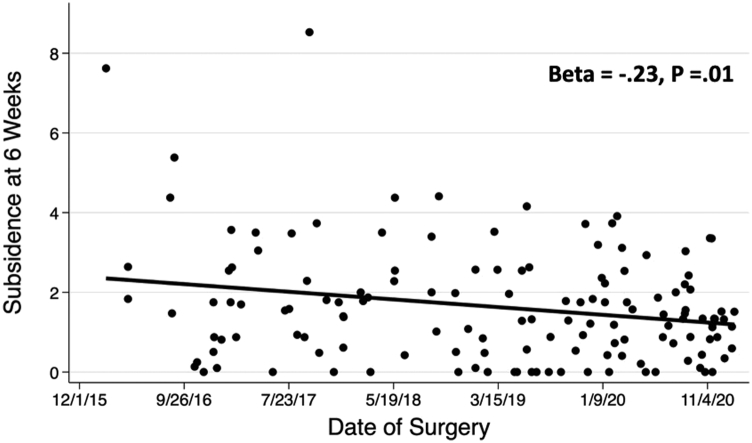
Early femoral component subsidence at 6 weeks post-operatively over time utilizing the direct anterior approach.

**Figure 4:**
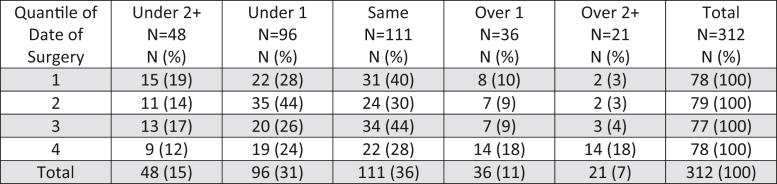
Implanted stem size compared to templated stem size for the posterior approach.

**Figure 5:**
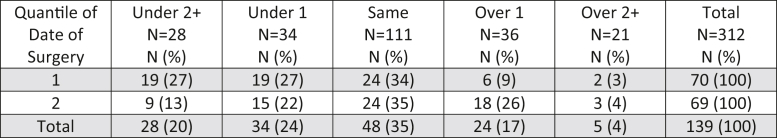
Implanted stem size compared to templated stem size for the direct anterior approach.

A total of 451 patients met inclusion criteria and were analyzed (Table 1). When stratified by the posterior approach, there was no association between the date of surgery and early femoral component subsidence (*P*-value for linear trend over time = .44; Fig. 2). With the direct anterior approach, date of surgery was inversely associated with statistically greater early femoral component subsidence (*P*-value for linear trend over time = .01; Fig. 3). Over time, in both posterior and anterior approaches, the surgeon was statistically more likely to implant a larger size stem compared to what was templated (*P*-value <.001 and Fig. 4; *P* = .03 and Fig. 5, respectively).

Twelve patients underwent reoperation within 90 days of their index procedure. Within this group, 2 underwent revision for instability, 3 for procedures related to infection, and 7 for periprosthetic fracture. Overall revision rate and incidence of periprosthetic fracture were independent of surgical approach (*P* = .20 and *P* = .21, respectively). There was no association between date of surgery and risk of 90-day reoperation (*P* = .45). None of the 451 patients underwent early revision for aseptic loosening.

## Discussion

In a surgeon’s initial use of a cementless tapered wedge stem design, there was no association between the date of surgery and early femoral component subsidence when the posterior approach was employed. When stratified based on direct anterior approach, there was an association between date of surgery and early femoral component subsidence. For either approach, however, no increased risk of 90-day reoperation was observed based on the date of surgery. Over time, the surgeon implanted a larger size stem relative to what was templated. No case of aseptic loosening was observed in any of the 451 patients.

Subsidence is a well-known complication involving cementless femoral component fixation in THA [12,13] and can be related to stem design [5,14]. Depending on the magnitude of subsidence, this can have implications on the long-term outcome of the prosthesis [15]. Magnitude of subsidence greater than or equal to 2-3 millimeters has been shown to correlate with worse clinical outcomes [10,[16], [17], [18]]. The majority of subsidence is believed to occur within the early postoperative period, specifically between the first 4-6 weeks [8,10]. In this study, early femoral component subsidence was not related to date of surgery during a surgeon’s learning curve with the implant via a posterior approach. An association was observed when the direct anterior approach was employed. As the surgeon’s initial use of this implant overlapped with his learning curve of the direct anterior approach, it is possible that the date of surgery and the surgeon’s personal experience with the direct anterior approach could confound each other’s association with subsidence. Further research is needed to better delineate this.

Interestingly, as surgeon experience with this tapered wedge stem grew, there was a tendency to implant a larger stem when compared to the preoperative templated size. This occurred regardless of surgical approach. Neither the tendency to implant a smaller stem early during the learning curve, nor the tendency to implant a larger size later in the learning curve was accompanied with increased risk of revision. This may be related, in part, to the inherent axial stability of a tapered wedge stem design [10]. Additionally, insertionally loose cementless stems have demonstrated the ability to subside and become rotationally stable with loading without decreased load to failure when compared to a well-fixed implant [19]. Previous literature in which examined fully hydroxyapatite-coated stems, like the implant examined in this study, did not find subsidence to be related to proximal femoral morphology [[20], [21], [22]]. These authors concluded that such stems are viable options for use in patients with Dorr type C proximal femurs. This further highlights the need for the surgeon to have a comprehensive understanding of the design characteristics of the utilized stem. The currently examined stem is designed to be line-to-line press fit, whereas this contrasts with other stems whose coating is designed to allow the stem to sit above the neck cut by 1-2 millimeters.

Much has been reported regarding the learning curve in the field of surgery. Subramonian and Muir [23] defined the learning curve as “the time taken and/or the number of procedures an average surgeon needs to be able to perform a procedure independently with a reasonable outcome.” This has inevitably extended to the realm of arthroplasty, most notably with the examination of the learning curve associated with THA performed via the direct anterior approach with estimates ranging from 20 to 100 cases [[24], [25], [26], [27]]. When studies examine the learning curve associated with a specific hip prosthesis, the majority of complications and need for revision procedures are found to occur during the learning curve associated with the implant. Eighty percent of periprosthetic fractures were seen during the first 30 surgical cases in primary THA case utilizing a novel stem [28]. Similarly, Khemka et al [29] found a 25-case learning curve for appropriate implant sizing and risk of intraoperative periprosthetic fracture when utilizing a neck-preserving stem.

Providers select and change implant brands for a variety of reasons. When practicing American Association of Hip and Knee Surgeons members were surveyed, Sharkey et al [30] found that the most frequently cited factors influencing implant choice were cost of components and anticipated improvement in clinical results. A similar study surveying fellowship-trained arthroplasty surgeons by Moss et al [31] reported that experience with the implant in fellowship or residency was most influential in implant selection. This was followed by contracts with their current employment institution, personal choice, and relationships with individual company sales representatives. For providers switching implant brands or former trainees beginning a new practice, it is important to consider that patients exposed early to a newly adopted technology may be at higher risk for complications until the learning curve is overcome [32]. In an age of increasing medical spending, these complications are often not without significant cost implications. Cumulative revision risk within 90 days of total hip arthroplasty has been reported to be 30.8%, and an absolute reduction of 1% would equate to a Medicare savings of nearly $1 billion over a 10-year period [33]. Moreover, prevention of a single periprosthetic fracture necessitating open reduction and internal fixation and/or revision arthroplasty would save at least $16,000 in readmission costs [34]. Our findings demonstrate a forgiving, cementless hip prosthesis that avoids increased adverse events in primary THA during the learning curve when compared to the surgeon’s most recent cases utilizing the same implant.

This study has several limitations, including those inherent to any retrospective review. The results of the study may be subject to confounding variable(s) that were not specifically measured. This study examined the initial experience of a single, fellowship-trained arthroplasty surgeon with a collarless, tapered wedge cementless femoral component. There was not a comparison group in which a different cementless femoral component was utilized. Previous work involving a different tapered wedge femoral component demonstrated that a collar provides significantly greater immediate stability when compared to a collarless form of the same implant [35]. Furthermore, collared varieties of this implant were observed to prevent significant subsidence [36]. As only the collarless form of the POLARSTEM implant was examined in this study, future work comparing our results to its collared counterpart is warranted. As previously mentioned, it is possible that the date of surgery and the surgeon’s personal experience with the direct anterior approach could confound each other’s association with subsidence. Despite a high intraobserver intraclass coefficient, radiographic measurements of subsidence are inevitably subject to some variation. Finally, the number of cases that defines the learning curve for a given implant is poorly defined and is subject to both the outcome measures as well as the specific implant under assessment. Based on recent practice trends, however, at least 30% of American Association of Hip and Knee Surgeons members perform 100 or fewer THAs annually [37].

## Conclusions

In a single surgeon’s initial use of a tapered cementless wedge stem, early femoral component subsidence was not impacted by the surgeon’s learning curve when the posterior approach was employed. Although subsidence was associated with date of surgery in the direct anterior cohort, neither approach was associated with an increased risk of 90-day reoperation. Should a surgeon choose to adopt a new femoral stem, these findings suggest that the stem is forgiving both in relation to subsidence and 90-day reoperation risk when appropriate surgical technique is utilized.

Conflict of interests

Stephen T. Duncan is a board member of BOC; received research support from Smith and Nephew, Medtronic, BoneSupport, Stryker, and Zimmer/Biomet; has stock options in MiCare and ROMTech; is a paid consultant for Smith and Nephew, BoneSupport,and OrthAlign; and is in speakers bureau for Smith and Nephew, BoneSupport,and OrthAlign. Cale Jacobs is a board member of ORS; is a part of Video Journal of Sports Medicine medical/orthopaedic publications editorial/governing board; and received research support from Smith and Nephew and Fexion Therapeutics. David Landy is part of AMJ Sports Med medical/orthopaedic publications editorial/governing board. All other authors declare no potential conflicts of interest.

For full disclosure statements refer to https://doi.org/10.1016/j.artd.2023.101119.

## Appendix A Supplementary Data

Conflict of Interest Statement for Duncan

Conflict of Interest Statement for Jacobs

Conflict of Interest Statement for Kluemper

Conflict of Interest Statement for Muffly

Conflict of Interest Statement for Landy
