# Supplementary material for: Penalty for Switching Implants? Assessing the Learning Curve With a Collarless, Tapered Wedge Cementless Femoral Component
Source: Arthroplast Today. 2023 Mar 6;20:101119. doi: 10.1016/j.artd.2023.101119 (PMC10009679; doi:10.1016/j.artd.2023.101119)
Supplement: Conflict of Interest Statement for Jacobs [file mmc2.pdf]

INDIVIDUAL CONFLICT OF INTEREST STATEMENT

American Association of Hip and Knee Surgeons

(Adopted from the American Academy of Orthopaedic Surgeons disclosure statement)

The following form **must be filled out completely and submitted by each author (example, 6 authors, 6 forms). All items require a response. If there is no relevant disclosure for a given item, enter "*None.*"**

Title: Penalty for Switching Implants? Assessing the Learning Curve with a Collarless, Tapered Wedge Cementless Femoral Component

1. Royalties from a company or supplier (The following conflicts were disclosed)
None

2. Speakers bureau/paid presentations for a company or supplier (The following conflicts were disclosed)
None

3A. Paid employee for a company or supplier (The following conflicts were disclosed)
None

3B. Paid consultant for a company or supplier (The following conflicts were disclosed)
None

3C. Unpaid consultants for a company or supplier (The following conflicts were disclosed)
None

4. Stock or stock options in a company or supplier (The following conflicts were disclosed)
None

5. Research support from a company or supplier as a Principal Investigator (The following conflicts were disclosed)
Smith & Nephew, Flexion therapeutics

6. Other financial or material support from a company or supplier (The following conflicts were disclosed)
None

7. Royalties, financial or material support from publishers (The following conflicts were disclosed)
None

8. Medical/Orthopaedic publications editorial/governing board (The following conflicts were disclosed)
Video Journal of Sports Medicine

9. Board member/committee appointments for a society (The following conflicts were disclosed)
ORS

Each author must sign AND print or type his/her name, date and submit a separate form

In addition, one BLINDED Conflict of Interest form (no author names used) should be submitted per manuscript with all author disclosures.

Cale Jacobs, PhD	[signature]	7/1/22
Author Name (Print or Type)	Author Signature	Date
